# A quantitative trait variant in *Gabra2* underlies increased methamphetamine stimulant sensitivity

**DOI:** 10.1111/gbb.12774

**Published:** 2021-10-22

**Authors:** Lisa R. Goldberg, Emily J. Yao, Julia C. Kelliher, Eric R. Reed, Jiayi Wu Cox, Cory Parks, Stacey L. Kirkpatrick, Jacob A. Beierle, Melanie M. Chen, William E. Johnson, Gregg E. Homanics, Robert W. Williams, Camron D. Bryant, Megan K. Mulligan

**Affiliations:** 1 Laboratory of Addiction Genetics, Department of Pharmacology and Experimental Therapeutics and Psychiatry, Boston, Massachusetts, USA; 2 NIGMS T32 Ph.D. Training Program in Biomolecular Pharmacology, Boston University School of Medicine, Boston, Massachusetts, USA; 3 Ph.D. Program in Bioinformatics, Boston University, Boston, Massachusetts, USA; 4 Program in Biomedical Sciences, Graduate Program in Genetics and Genomics, Boston University School of Medicine, Boston, Massachusetts, USA; 5 Department of Agricultural, Biology, and Health Sciences, Cameron University, Lawton, Oklahoma, USA; 6 Department of Medicine, Computational Medicine, Boston University School of Medicine, Boston, Massachusetts, USA; 7 Departments of Anesthesiology, Neurobiology, and Pharmacology and Chemical Biology, University of Pittsburgh, Pittsburgh, Pennsylvania, USA; 8 Department of Genetics, Genomics and Informatics, University of Tennessee Health Science Center, Memphis, Tennessee, USA

**Keywords:** addiction, amphetamine, cocaine, eQTL, methylphenidate, psychostimulant, quantitative trait gene, quantitative trait nucleotide, stimulant disorders

## Abstract

Psychostimulant (methamphetamine, cocaine) use disorders have a genetic component that remains mostly unknown. We conducted genome-wide quantitative trait locus (QTL) analysis of methamphetamine stimulant sensitivity. To facilitate gene identification, we employed a Reduced Complexity Cross between closely related C57BL/6 mouse substrains and examined maximum speed and distance traveled over 30 min following methamphetamine (2 mg/kg, i.p.). For maximum methamphetamine-induced speed following the second and third administration, we identified a single genome-wide significant QTL on chromosome 11 that peaked near the *Cyfip2* locus (LOD = 3.5, 4.2; peak = 21 cM [36 Mb]). For methamphetamine-induced distance traveled following the first and second administration, we identified a genome-wide significant QTL on chromosome 5 that peaked near a functional intronic indel in *Gabra2* coding for the alpha-2 subunit of the GABA-A receptor (LOD = 3.6–5.2; peak = 34–35 cM [66–67 Mb]). Striatal *cis*-expression QTL mapping corroborated *Gabra2* as a functional candidate gene underlying methamphetamine-induced distance traveled. CRISPR/Cas9-mediated correction of the mutant intronic deletion on the C57BL/6J background to the wild-type C57BL/6NJ allele was sufficient to reduce methamphetamine-induced locomotor activity toward the wild-type C57BL/6NJ-like level, thus validating the quantitative trait variant (QTV). These studies show the power and efficiency of Reduced Complexity Crosses in identifying causal variants underlying complex traits. Functionally restoring *Gabra2* expression decreased methamphetamine stimulant sensitivity and supports preclinical and human genetic studies implicating the GABA-A receptor in psychostimulant addiction-relevant traits. Importantly, our findings have major implications for studying psychostimulants in the C57BL/6J strain—the gold standard strain in biomedical research.

## 1 | INTRODUCTION

Psychostimulant (methamphetamine, cocaine) use disorders (PUDs) are a serious public health concern. Until the COVID-19 pandemic emerged, the opioid epidemic crisis had begun to plateau. Meanwhile, PUDs have quietly made a resurgence, with increased use and deaths.^1,2^ Yet, despite an estimated 40%–50% heritability for PUDs,^3–5^ genome-wide association studies have identified few loci.^6^ In one study, a significant GWAS hit for cocaine dependence mapped to *FAM53B*.^6,7^ Notably, an unbiased, quantitative trait locus (QTL) approach in mice identified a *trans*-expression QTL regulating *Fam53b* expression that was genetically correlated with variance in cocaine intravenous self-administration (IVSA) in BXD-RI mice, exemplifying cross-species bidirectional translation with discovery genetics in rodents.

*Reduced Complexity Crosses* exploit the extreme, near-isogenic nature of closely related inbred substrains to rapidly map, pinpoint and validate quantitative trait loci (QTLs) containing causal quantitative trait genes (QTGs) and quantitative trait variants (QTVs) underlying complex trait variation,^8,9^ including gene expression and behavior.^10–13^ Of relevance to the present study, Kumar and colleagues used a mouse Reduced Complexity Cross between C57BL/6 substrains to map a missense variant in *Cyfip2* with sensitivity to cocaine-induced velocity and extended these findings to methamphetamine.^12^ We previously used a similar Reduced Complexity Cross to map and validate *Cyfip2* in binge-like eating.^11^ Also of relevance to the present study, we exploited the reduced complexity of C57BL/6 substrains to identify a functional noncoding single nucleotide deletion in *Gabra2* (alpha-2 subunit of the GABA-A receptor) that induced a loss-of-function decrease in transcript and protein expression.^13^ Correction of this mutation via CRISPR/Cas9 gene editing restored Gabra2 expression at both the transcript and protein levels.^13^ DBA/2 mouse substrains combined with historical BXD-RI substrains have also been exploited to identify a functional missense variant in trace amine-associated receptor 1 (*Taar1*) underlying differences in the aversive properties of methamphetamine self-administration, body temperature and toxicity.^14–17^

Administration of addictive drugs such as opioids and psychostimulants increases dopamine release in forebrain regions, including the dorsal striatum and nucleus accumbens, which contributes to the locomotor stimulant and rewarding properties of drugs of abuse.^18,19^ Psychostimulant-induced locomotor activity is a rapid, high-throughput heritable trait that is amenable to QTL mapping in multiple genetic populations^20–23^ and has a shared genetic basis with other addiction-relevant behavioral traits. As two examples, we mapped and validated genetic factors influencing psychostimulant and opioid-induced locomotor activity, including *Csnk1e*^24^ and *Hnrnph1*.^25^ Subsequently, we and others have extended the role of these two genes to other complex behavioral models for addiction, including reward as measured via conditioned place preference^26,27^ and reinforcement as measured via intravenous and oral self-administration.^27,28^

C57BL/6J (B6J) and C57BL/6NJ (B6NJ) are two substrains of C57BL/6, the most commonly used mouse strain in biomedical research, and are 99.9% genetically similar, yet exhibit significant differences in several addiction-associated traits,^29^ including ethanol consumption,^30,31^ nicotine behaviors^32^ and psychostimulant behaviors.^12,29^ Although phenotypic differences between B6 substrains can be quite large, genotypic diversity is extremely small, with only an estimated 10,000 to 20,000 variants (SNPs plus indels) distinguishing the two strains.^33–35^

In the present study, we used a Reduced Complexity Cross between C57BL/6 substrains to map the genetic basis of sensitivity to the locomotor stimulant properties of methamphetamine, including maximum speed and distance traveled. Following the identification of two historical loci, including one locus for sensitized methamphetamine-induced maximum speed near the *Cyfip2* missense mutation that was previously identified for acute and sensitized cocaine velocity^12^ and a second locus near the functional intronic variant in *Gabra2*,^13^ we used a CRISPR/Cas9 gene-edited knockin mouse model with the corrected *Gabra2* mutation to validate this functional indel as necessary for enhanced acute stimulant sensitivity that is exhibited in the parental C57BL/6J substrain.^12^

## 2 | MATERIALS AND METHODS

### 2.1 | C57BL/6J (B6J), C57BL/6NJ (B6NJ) and a B6J × B6NJ-F2 reduced complexity cross (Bryant Lab, BUSM)

All experiments involving mice were approved by the Boston University School of Medicine (BUSM) and University of Tennessee Health Science Center (UTHSC) Institutional Animal Use and Care Committees and were conducted in accordance with the AAALAC Guide for the Use and Care of Laboratory Animals.^36^ Mice were housed in an AAALAC-accredited temperature- and climate-controlled facilities on a 12 h light/dark cycle (lights on at 0630 h for BUSM and 0600 h for UTHSC). Mice were housed in same-sex groups of two to five mice per cage with standard laboratory chow and water available ad libitum except during testing. All behavioral testing was performed during the light phase of the 12 h light/dark cycle.

C57BL/6J mice (**B6J**; n = 31; 16 females, 15 males; all 68 days old on Day 1 the 5-day locomotor protocol) and C57BL/NJ mice (**B6NJ**; n = 32; 16 females, 16 males, all 68 days old on Day 1 of the 5-day locomotor protocol) were purchased from The Jackson Laboratory (Bar Harbor, ME) at 7 weeks of age and were habituated in the vivarium 1 week prior to experimental testing that occurred next door. For QTL mapping, a unidirectional cross was conducted whereby B6J females were crossed to B6NJ males to generate B6J × B6NJ-F_1_ mice and B6J × B6NJ F_1_ offspring were intercrossed to generate B6J × B6NJ F_2_ mice. All mice within a cage were assigned the same treatment.

All mice comprising the parental substrains and F2 offspring for which we present behavioral data had a prior, identical history of naloxone-induced conditioned place aversion as described in our original publication.^37^ Notably, however, the parental substrain breeders and F1 breeders that were used to generate the phenotyped F2 offspring were not themselves phenotyped and were experimentally naive. F2 females and F2 males were 62–114 days old on Day 1 of the five-day locomotor protocol. Briefly, following initial assessment of preference for the drug-paired side, 24 h later, mice received two alternating injections of naloxone hydrochloride (4 mg/kg, i.p.) and two alternating injections of saline (i.p.), separated by 48 h. Then, 72 and 96 h after the second saline trial, mice were re-assessed for drug-free and state-dependent conditioned place aversion for the naloxone-paired side, respectively. Thus, all mice received a total of three injections of 4 mg/kg naloxone over 9 days. One week following recovery from the test for naloxone-induced conditioned place aversion, mice were tested for methamphetamine stimulant sensitivity in a five-day protocol (two saline injections, three methamphetamine injections) as described below.

### 2.2 | *Gabra2* knockin mice

Gene-edited knockin mice were generated by inserting the corrected *Gabra2* single intronic nucleotide on the mutant C57BL/6J (B6J) background via CRISPR/Cas9 gene editing as previously described.^13^ Between 1976 and 1991, this single nucleotide deletion became fixed and exists in many engineered lines, BXD recombinant inbred strains that were generated after 1991, the Collaborative Cross, Diversity Outbred mice and the majority of consomic lines.^13^ The C57BL/6NJ (B6NJ) substrain contains the deleted nucleotide and thus, inserting the nucleotide into the genome of the B6J background is predicted to modulate Gabra2-related phenotypes in a direction consistent with the B6NJ phenotype. Briefly, a sgRNA was designed that targeted *Gabra2* at the intron/exon junction near chromosome 5 at 71,014,638 Mb (mm10). A T7 promoter containing the sgRNA template was used to produce sgRNA and Cas9 mRNA that was then purified, ethanol precipitated and re-suspended in DEPC-treated water. A 121 nucleotide single-stranded DNA repair template oligo with the T insertion in the intron of *Gabra2* along with sgRNA and *Cas9* mRNA were co-injected into the cytoplasm of B6J one-cell embryos. Offspring of injected embryos were screened for the insertion via PCR amplification of the knockin site. PCR products containing the amplicon were sequenced directly or subcloned into pCR2.1-TOPO (Invitrogen) and sequenced. The male founder (F0) was crossed to female B6J mice to generate F1 progeny. F1 mice were crossed to generate F2 mice. The colony is maintained through heterozygous breeding and all behavioral phenotyping was performed in generations F2 and higher. Potential off-targets were screened using CRISPOR (RRID:SCR_015935)^38,39^ as previously described^13^ and no off-target modifications were detected in the top 15 predicted off-target sites. A total of 8 wild-type females (91–155 days old), 10 wild-type males (99–121 days old), 8 KI females (91–149 days old) and 9 KI males (86–149 days old) were tested. Age ranges reflect the age on Day 1 of the five-day locomotor protocol.

### 2.3 | Drugs

Methamphetamine hydrochloride (MA) (Sigma, St. Louis, MO) was dissolved in sterilized, physiological saline (0.9%) prior to injection (10 ml/kg, i.p.). The dose of MA (2 mg/kg) was chosen based on our prior success in mapping QTLs with this dose^24,25,40,41^ and based on a previous study that identified C57BL/6 substrain differences in MA-induced locomotor activity.^12^

### 2.4 | Methamphetamine-induced maximum speed and distance traveled in B6J, B6NJ and B6J × B6NJ-F2 mice (Bryant Lab, BUSM)

The plexiglas apparatus consisted of an open field (40 cm length × 20 cm width × 45 cm tall; Lafayette Instruments, Lafayette, IN) surrounded by a sound-attenuating chamber (Med Associates, St. Albans, VT) that was unlit inside. Behaviors were recorded using a security camera system (Swann Communications, Melbourne, Australia) and then video tracked (Anymaze, Stoelting, Wood Dale, IL). We employed a 5-day locomotor protocol,^41^ which is an extended version of the 3-day protocol comprising the acute methamphetamine response.^25^ On Days (D)1 and 2, following habituation to the next-door testing room for a minimum of 30 min, mice were injected with SAL (100 ul/10 g, i.p.). On D3, D4 and D5 mice were injected with methamphetamine (2 mg/kg, 100 ul/10 g, i.p.). Following i.p. injection, mice were immediately placed into the open field and video recorded over 30 min. Each mouse was tested at the same time of day, every day until completion of the 5-day protocol. Locomotor phenotypes, including total distance traveled and maximum speed while mobile, were calculated with AnyMaze.

Data were analyzed using repeated measures ANOVA with strain and sex as factors and day as a categorical repeated measure. Time course analysis for a given day was analyzed in a similar manner but with Time (six, 5-min bins) as the repeated measure. Post hoc analysis was conducted using the family-wide error variance with simple contrasts and Tukey’s honestly significant difference to correct for multiple comparisons. Mice were tested during the light phase of the 12/12 h light/dark cycle (lights on at 0630 h) between 0800 and 1300 h. Each mouse was tested at the same time of day, every day, until completion of the 5-day protocol.

### 2.5 | Methamphetamine-induced distance traveled in Gabra2 knockin (KI) mice (Mulligan Lab, UTHSC)

Validation for the role of the *Gabra2* functional intronic single nucleotide deletion (see following text) in methamphetamine-induced distance traveled was conducted in the Mulligan Lab at UTHSC. Procedures were similar, although not identical. The regimen was identical (saline on Days 1–2; 2 mg/kg methamphetamine on Days 3–5). The major difference was that a larger open field arena (40 cm × 40 cm × 40 cm) was employed at UTHSC and there was no sound-attenuating chamber. Also, unlike the arenas at BUSM, the UTHSC arenas were open air arenas without sound attenuating chambers. Additionally, mice were tail marked and handled for at least 3 days prior to testing. Prior to injection, the experimenter examined the tail marks and placed each mouse in a separate holding cage with clean bedding for at least 5 min before receiving an injection of saline (100 μl per 30 g body weight, i.p.). i.p.) or methamphetamine (2 mg/kg, i.p.) or saline. All other procedures were the same as BUSM. The larger arena size (1.5-fold larger than the arena used in the Bryant Lab) likely accounts for the overall higher level of locomotor activity in this study compared with the parental strain and F2 studies. Similar to the Bryant Lab, behavior was also recorded with a security camera system and locomotor phenotypes were also calculated in AnyMaze. Mice were also tested during the light phase of the 12 h/12 h light/dark cycle (lights on at 0600 am) between 1000 and 1400 h. Each mouse was tested at the same time of day, every day, until completion of the 5-day protocol.

### 2.6 | Statistical analysis of parental substrains and KI mice

In analyzing summed data across days, we first ran repeated measures (RM) analysis of variance (ANOVA) with Substrain/Genotype and Sex as factors and Day as the repeated measure. We then ran separate RM ANOVAs for females and males with Substrain/Genotype as a factor and Day as the repeated measure. In analyzing time course data post-methamphetamine, similar analyses were conducted, except that “Time” (5-min bins) rather than Day was treated as the repeated measure. Tukey’s post hoc or simple contrasts were conducted to identify the source of main effects and interactions.

### 2.7 | DNA collection and genotyping in B6J × B6NJ-F2 mice

DNA was extracted from spleens of F2 mice and prepared for genotyping using a standard salting out protocol. Ninety SNP markers spaced ~30 Mb (~15 cM) apart were genotyped using a custom-designed 96 × 96 Fluidigm SNPtype array (South San Francisco, CA).^10,11^ SNPs were called using the Fluidigm SNP Genotyping Analysis Software and SNPtype normalization with the default threshold.

### 2.8 | QTL analysis in B6J × B6NJ-F2 mice

QTL analysis was performed in F_2_ mice using the R package R/qtl (RRID:SCR_009085) as previously described.^11,29,42^ Quality checking of genotypes and QTL analysis were performed in R (https://www.r-project.org/) using R/bestNormalize (https://github.com/petersonR/bestNormalize) and R/qtl.^42^ Phenotypes were assessed for normality using the Shapiro–Wilk Test. Because some of the data residuals deviated significantly from normality, we used the orderNorm function to perform Ordered Quantile normalization^43^ on all phenotypes. QTL analysis was performed using the “scanone” function and Haley-Knott (HK) regression. Permutation analysis (perm = 1000) was used to compute genome-wide suggestive (*p* < 0.63) and significance thresholds for log of the odds (LOD) scores (*p* < 0.05). Sex was included as an additive covariate in the QTL model. In a follow-up analysis, Age was also included as a covariate. Peak marker positions were converted from sex-averaged cM to Mb using the JAX Mouse Map Converter (http://cgd.jax.org/mousemapconverter). Percent phenotypic variance explained by each QTL was calculated using the “fitqtl” function.

Power analysis of Day 5 maximum speed and Day 3 distance traveled using the data from 184 F2 mice was conducted using the R package R/qtlDESIGN (RRID:SCR_013424).^44^ For each behavioral phenotype, we generated plots showing power versus % variance explained for an additively inherited QTL.

### 2.9 | RNA-seq

Striatum was chosen for eQTL analysis for historical reasons and because this brain region is a major local site of drug action where methamphetamine binds to the dopamine transporter (and other monoamine transporters) and vesicular monoamine transporters to cause reverse transport of dopamine into the synapse, thus inducing stimulant, rewarding and reinforcing effects.^45^ Furthermore, Gabra2-containing GABA-A receptors are concentrated in the striatum^46^ and are a dominant receptor type.^47^ Thus, the striatum is a highly relevant brain tissue to ascertain the effects of *Gabra2* genetic variation on the transcriptome as they relate to methamphetamine-induced locomotor stimulation.

Striatum punches were collected as described^25^ for RNA-seq from 23 F2 mice. Details on the prior experimental history of these mice are published.^10^ Mice were previously trained and tested for place conditioning to oxycodone hydrochloride (1.25 mg/kg, i.p.; a total of 3 injections over 9 days). Six days later, these mice underwent an additional 4 daily injections of oxycodone (20 mg/kg, i.p.) before being tested for antinociception on the hot plate and then the following week, an additional 4 daily injections of the same dose of oxycodone prior to testing for affective withdrawal on the elevated plus maze 16 h later. Brains were harvested 24 h after behavioral assessment of oxycodone withdrawal (~40 h after the final injection of oxycodone).

Brains were rapidly removed and sectioned with a brain matrix to obtain a 3 mm thick section where a 2 mm diameter punch of the striatum was collected. Left and right striatum punches were pooled and immediately placed in RNA later (Life Technologies, Grand Island, NY) and stored for 48 h at 4°C prior to storage in a − 80°C freezer. Total RNA was extracted using the RNeasy kit (Qiagen, Valencia, CA) as described.^25^ RNA was shipped to the University of Chicago Genomics Core Facility for cDNA library preparation using the Illumina TruSeq (oligo-dT; 100 bp paired-end reads). Libraries were prepared according to Illumina’s detailed instructions accompanying the TruSeq^®^ Stranded mRNA LT Kit (Part# RS-122–2101). The purified cDNA was captured on an Illumina flow cell for cluster generation and sample libraries were sequenced at 23 samples per lane over five lanes (technical replicates) according to the manufacturer’s protocols on the Illumina HiSeq 4000 machine, yielding an average of 69.4 million paired-end reads per sample. FASTQ files were quality checked via FASTQC and possessed Phred quality scores >30 (i.e., less than 0.1% sequencing error). This data set is publicly available on Gene Expression Omnibus (GEO #119719).

### 2.10 | *Cis*- and *trans*-expression QTL (eQTL) analysis

Details on eQTL mapping are published.^10^ We conducted eQTL analysis on striatal samples from 23 mice (8 F2 females, 15 F2 males; age range = 80–128 days old at the time of tissue harvesting). We aligned FastQ files to the reference genome (mm38) via TopHat (RRID: SCR_013035)^48^ using the mm38 build and Ensembl Sequence and genome annotation. We used *featureCounts* (RRID:SCR_012919) to count and align reads. For *cis*-eQTL analysis, we used the same marker panel that we used in behavioral QTL analysis. We removed lowly expressed exons that did not possess at least 10 reads total across all 115 count files. Because of the low resolution of QTL mapping in an F2 cross, we liberally defined a gene with a *cis*-eQTL as any gene possessing a genome-wide significant association between expression and a polymorphic marker that was within 70 Mb of a SNP (the largest distance between any two SNPs from the 90-SNP panel). Analysis was conducted using *limma* (RRID:SCR_010943) with default TMM normalization and VOOM transformation.^49,50^ We used *limma* rather than R/qtl for eQTL analysis which allowed us to account for technical replicates in the analysis based on multiple sequencing of the same samples across sequencing runs. A linear model was employed whereby sample replicates were treated as a random effects repeated measure. The duplicate Correlation() function was used to estimate within-sample correlation, which was then included in the lmFit() function. An ANOVA test was conducted for gene expression that included Sex and Age as covariates and Genotype as a fixed effect. Gene-level tests were conducted using the likelihood Ratio test. A false discovery rate of 5% was employed as the cut-off for statistical significance.^51^

### 2.11 | Enrichment analysis

Enrichment analysis of genes whose transcripts correlated with Gabra2 transcript levels (*r* ≤ −0.5 or *r* ≥ 0.5; *p* ≤ 0.015) was conducted using the online tool Enrichr (RRID:SCR_001575)^52,53^ where we report GO terms for molecular, cellular and biological function.

## 3 | RESULTS

### 3.1 | B6J mice show an increase in methamphetamine-induced maximum speed and locomotor activity compared with B6NJ mice

**FIGURE 1:**
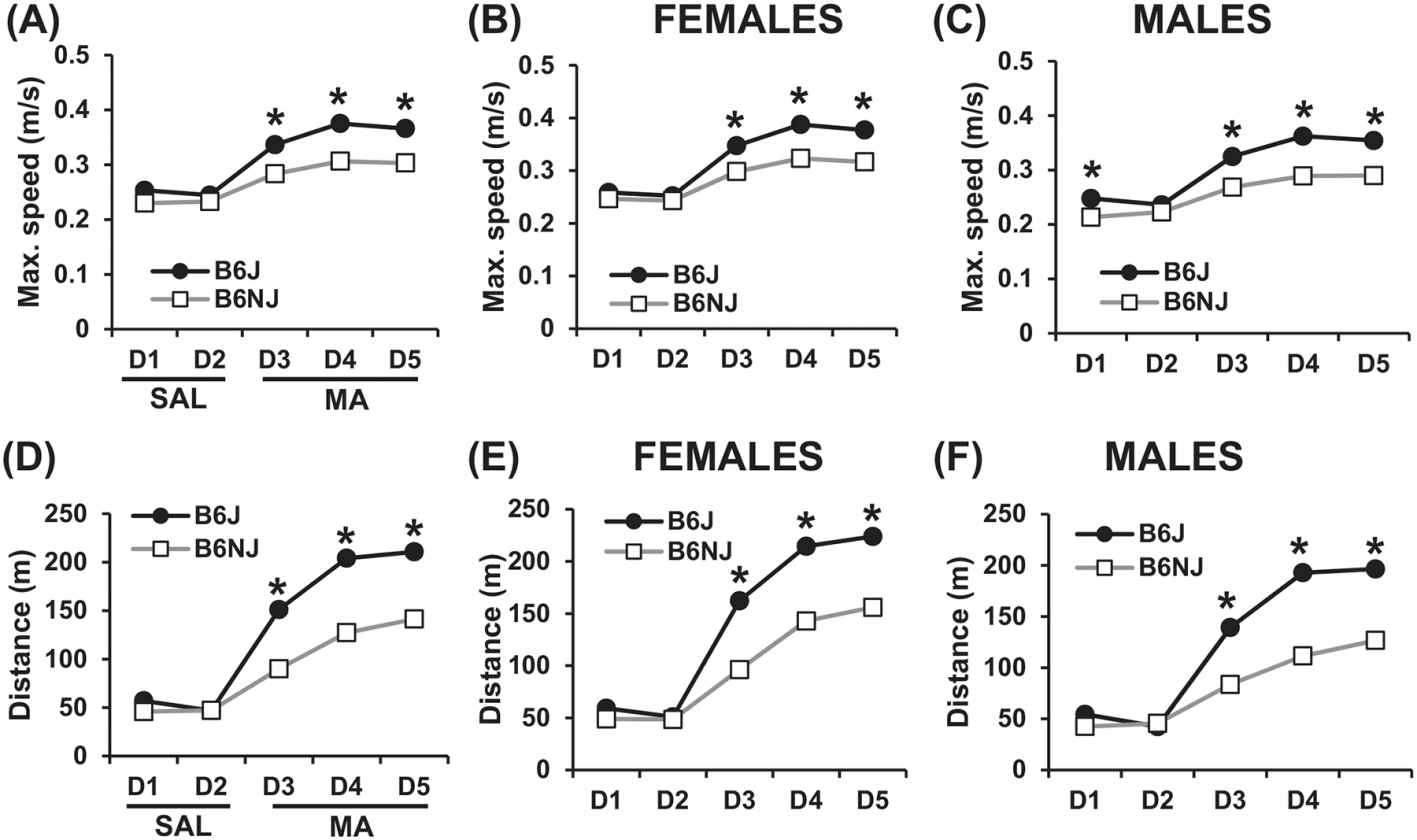
Maximum speed and distance traveled in response to saline (Days 1–2) and methamphetamine (Days 3–5) in the parental C57BL/6J (B6J) and C57BL/6NJ (B6NJ) substrains. (A) Sex-combined maximum speed (m/s) across training days. There was a significant increase in maximum speed in B6J versus B6NJ mice on Day(D) 3, D4 and D5 (*all *p*’s_adjusted_ < 0.0001). (B) Maximum speed (m/s) across training days in females. There was a significant difference increase in maximum speed in B6J versus B6NJ females on D3 (*p_adjusted_ = 0.0005), D4 and D5 (*p’s_adjusted_ < 0.0001). (C) Maximum speed across training days in males. There was a significant increase in maximum speed in B6J versus B6NJ males on D1 (*p*_adjusted_ = 0.03), D3, D4 and D5 (*p’s_adjusted_ < 0.0001). (D) Sex-combined distance traveled (m) across training days. There was a significant increase in distance traveled in B6J versus B6NJ mice on D3, D4 and D5 (all *p*’s_adjusted_ < 0.0001). (E) Distance traveled across training days in females. There was a significant increase in distance traveled in B6J versus B6NJ females on D3, D4 and D5 (*all *p*’s_adjusted_ < 0.0001). (F) Distance traveled across training days in males. There was a significant increase in distance traveled in B6J versus B6NJ males on Day 3, Day 4 and Day 5 (*all *p*’s_adjusted_ < 0.0001)

In examining B6 substrain differences in maximum speed, there were no substrain difference in response to saline injections (10 ml/kg, i.p.) on Days 1 and 2 (p’s > 0.05). However, in response to methamphetamine (2 mg/kg, i.p.) on Day 3, Day 4 and Day 5, B6J mice showed increased maximum speed relative to B6NJ mice, regardless of whether the data were collapsed across Sex or analyzed separately in females and males (Figure 1A–C). RM ANOVA of the sex-combined data set across days indicated a main effect of Substrain (F_1,59_ = 59.96, *p* = 1.49 × 10^−10^), Sex (F_1,59_ = 18.02, *p* = 7.85 × 10^−5^), Day (F_4,236_ = 377.90, *p* < 2 × 10^−16^) and a Substrain × Day interaction (F_4,236_ = 24.51, *p* < 2 × 10^−16^). Tukey’s post hoc showed a significant increase in maximum speed in B6J versus B6NJ mice on Day(D) 3, D4 and D5 (*all *p*’s_adjusted_ < 0.0001; Figure 1A). In breaking down the data set by Sex, for females-only, RM ANOVA indicated a main effect of Substrain (F_1,30_ = 22.29, *p* = 5.11 × 10^−5^), Day (F_4,120_ = 199.44, *p* < 2 × 10^−16^) and a Substrain × Day interaction (F_4,120_ = 13.21, *p* = 6 × 10^−9^). Tukey’s post hoc test showed a significant difference increase in maximum speed in B6J versus B6NJ females on D3 (*p_adjusted_ = 0.0005), D4 and D5 (*p’s_adjusted_ < 0.0001; Figure 1B). For males-only, RM ANOVA indicated a main effect of Substrain (F_1,29_ = 38.67, *p* = 8.75 × 10^−7^), Day (F_4,116_ = 178.66, *p* < 2 × 10^−16^) and a Substrain × Day interaction (F_4,116_ = 11.67, *p* = 5.36 × 10^−8^). Tukey’s post hoc showed a significant increase in maximum speed in B6J versus B6NJ males on D1 (*p*_adjusted_ = 0.03), D3, D4 and D5 (*p’s_adjusted_ < 0.0001; Figure 1C).

In examining B6 substrain differences in distance traveled following saline versus methamphetamine, there were no substrain differences in distance traveled in response to saline on Days 1 and 2 (*p*’s > 0.05). However, in response to methamphetamine, B6J mice showed an increase in distance traveled compared with B6NJ mice on Day 3, Day 4 and Day 5, regardless of whether the data were collapsed across Sex or analyzed separately in females and males (Figure 1D–F). RM ANOVA of the sex-combined data set across days showed a main effect of Substrain (F_1,59_ = 82.39, *p* = 8.51 × 10^−13^), Day (F_4,236_ = 798.0, *p* < 2 × 10^−16^), a Substrain × Day interaction (F_4,236_ = 67.99, *p* < 2 × 10^−16^) and a Sex × Day interaction (F_4,236_ = 6.46; *p* = 6.0 × 10^−5^). Tukey’s post hoc showed a significant increase in distance traveled in B6J versus B6NJ mice on D3, D4 and D5 (all *p*’s_adjusted_ < 0.0001; Figure 1D). In breaking down the data set by Sex, RM ANOVA of females-only indicated a main effect of Substrain (F_1,30_ = 41.18, *p* = 4.37 × 10^−7^), Day (F_4,120_ = 408,83, *p* < 2 × 10^−16^) and a Substrain × Day interaction (F_4,120_ = 27.05, *p* = 5.27 × 10^–10)^. Tukey’s post hoc showed a significant increase in distance traveled in B6J versus B6NJ females on D3, D4 and D5 (*all *p*’s_adjusted_ < 0.0001; Figure 1E). For males-only, RM ANOVA indicated a main effect of Substrain (F_1,29_ = 40.16, *p* = 6.34 × 10^−7^), Day (F_4,116_ = 396.98, *p* < 2 × 10^−16^) and a Substrain × Day interaction (F_4,116_ = 44.59, *p* < 2 × 10^−16^). Tukey’s post hoc test showed a significant increase in distance traveled in B6J versus B6NJ males on Day 3, Day 4 and Day 5 (*all *p*’s_adjusted_ < 0.0001; Figure 1F).

To summarize, these results replicate previous B6 substrain differences in the B6 mice from the J lineage show increased stimulant sensitivity compared with B6 mice from the N lineage.^12^

### 3.2 | Chromosome 11 QTL near the *Cyfip2* missense mutation underlying variation in sensitized methamphetamine-induced maximum speed (m/s)

**FIGURE 2:**
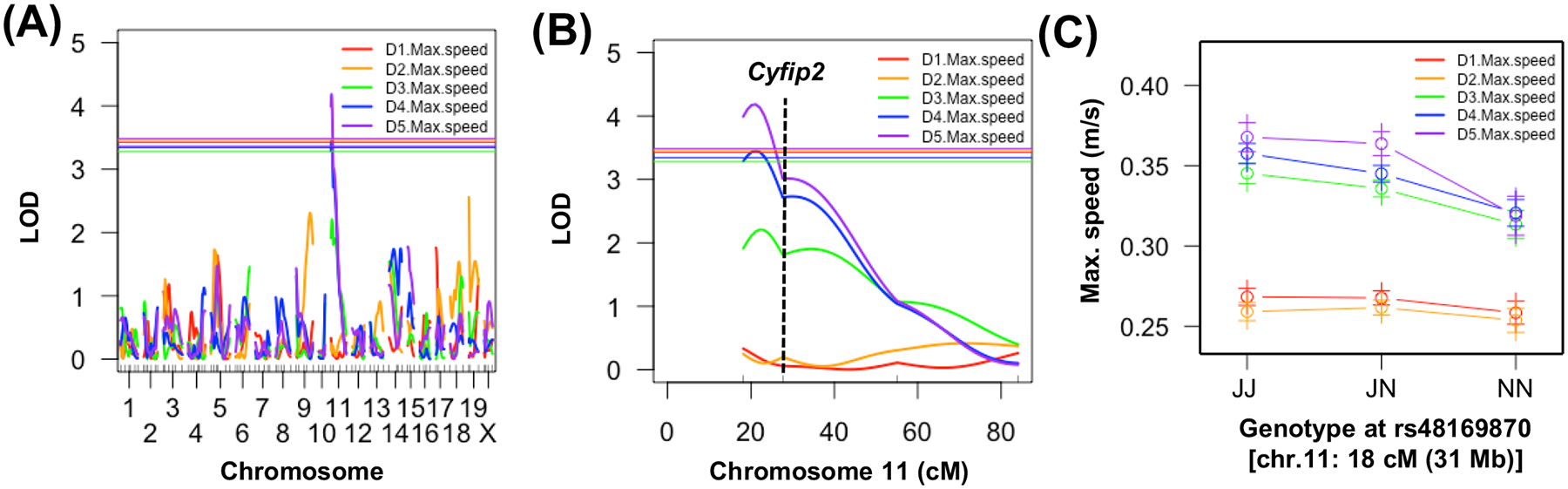
Genome-wide significant QTL on chromosome 11 near *Cyfip2* underlying variation in sensitized methamphetamine-induced maximum speed. Mice were treated on Day(D) 1 and D2 with saline (i.p.) and on D3, D4 and D5 with methamphetamine and behavioral activity was recorded over 30 min. (A) Genome-wide significant QTL on chromosome 11 for maximum speed following the second methamphetamine injection on D4 and following the third methamphetamine injection on D5. Solid horizontal lines for panels A and B indicate significance thresholds for each phenotype (*p* < 0.05). (B) Chromosome 11 QTL plot for maximum speed on D1 through D5. (C) Effect plot of maximum speed as a function of Genotype at the peak locus for maximum speed on D1 through D5. J, homozygous for B6J allele; BN, heterozygous; N, homozygous for B6NJ allele

Next, we sought to identify the genetic basis of differential methamphetamine-induced stimulant sensitivity in B6 substrains using an F2 Reduced Complexity Cross.^8,9^ We identified a genome-wide significant QTL on chromosome 11 underlying maximum speed while mobile that was specific to methamphetamine treatment and emerged after the second methamphetamine injection on Day 4 (LOD = 3.5; *p* = 0.039, peak = 21 cM [36 Mb]; Bayes interval: 18–39 cM [31–63 Mb]; 11% of the phenotypic variance explained; Figure 2A) and the third methamphetamine injection on Day 5 (LOD = 4.2, *p* = 0.009; peak = 21 cM [36 Mb]; Bayes interval: 18–34 cM [31–57 Mb]; 11% of the phenotypic variance explained; Figure 2A). The marker nearest the peak (rs48169870; 18 cM [31 Mb]) was located just proximally to the *Cyfip2* missense mutation (rs24064617401; 28 cM [46 Mb]; Figure 2B) previously identified for acute and sensitized cocaine velocity.^12^ Like the previous finding, the effect plot for maximum speed indicated that the B6J allele was associated with increased methamphetamine-induced maximum speed (Figure 2C). Thus, the locus containing the *Cyfip2* missense mutation is associated with behavioral sensitivity to multiple psychostimulants.

### 3.3 | Chromosome 5 QTL near the *Gabra2* intronic deletion underlying variation in acute methamphetamine-induced locomotor activity (total distance traveled; m)

**FIGURE 3:**
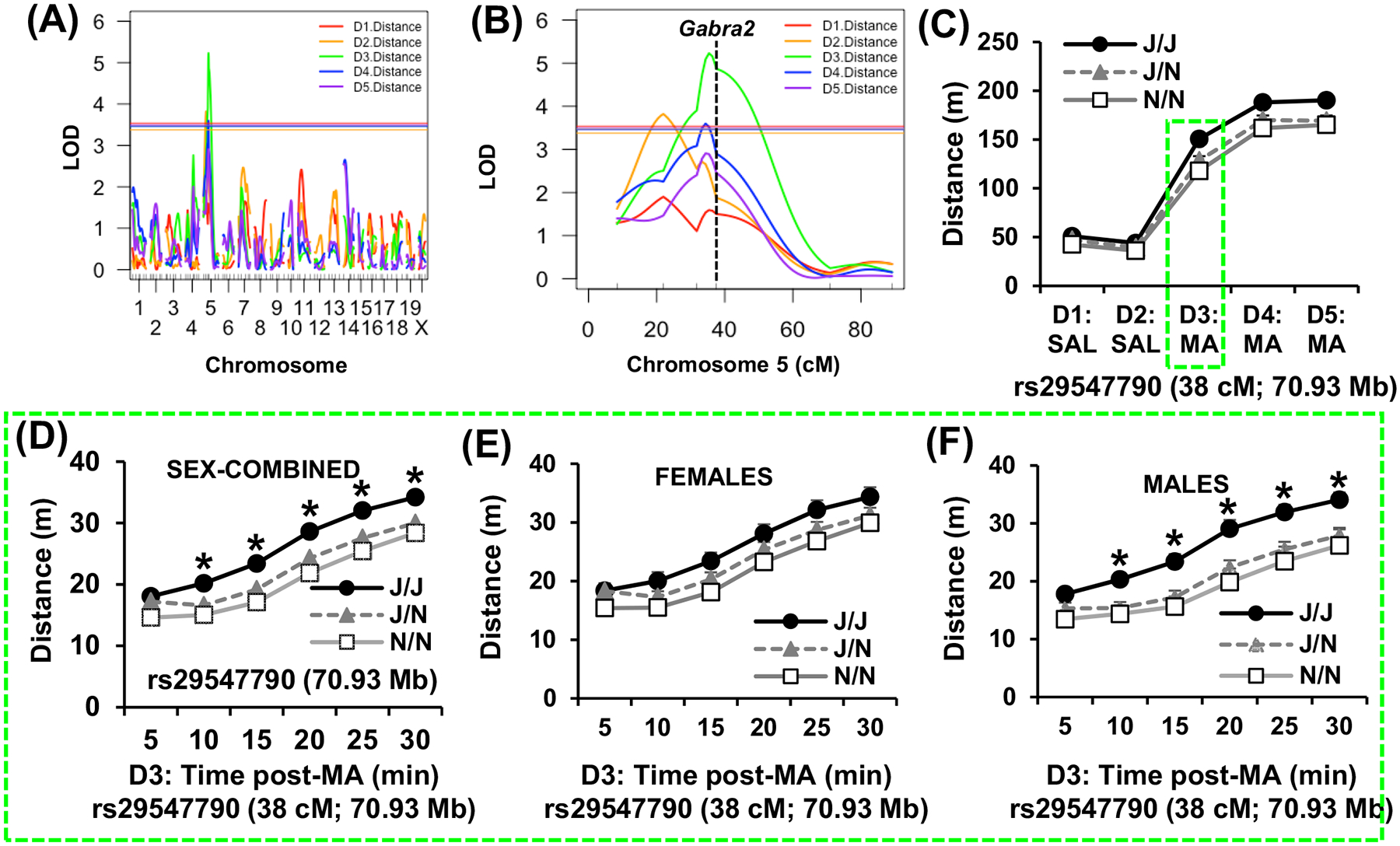
Genome-wide significant QTL on chromosome 5 near *Gabra2* underlying variation in acute methamphetamine-induced distance traveled. (A) Genome-wide significant QTL on chromosome 5 for distance traveled (m) on Day(D) 2 over 30 min following i.p. saline, a second QTL for distance traveled D3 following 2 mg/kg methamphetamine, and a third, similarly localized QTL on D4 following the second methamphetamine injection. Solid horizontal lines for panels A and B indicate significance threshold for each phenotype (*p* < 0.05). (B) Chromosome 5 QTL plot for distance traveled on D1 through D5. (C) Effect plot of total distance traveled for D1 through D5 over 30 min at the peak associated marker (rs29547790; 70.93 Mb). (D) Time course in 5-min bins of the effect plot for acute methamphetamine-induced distance traveled on D3. * = significant increase in J/J relative J/N and N/N (Tukey’s *p*_adjusted_ < 0.05 for the three comparisons at each time point). (E) Time-course in females. (F) Time-course in males. * = significant increase in J/J relative J/N and N/N (Tukey’s *p*_adjusted_ < 0.05 for the three comparisons at each time point). J = homozygous for B6J allele; BN = heterozygous; N = homozygous for B6NJ allele. Green, dashed traces denote distance traveled on D3

We identified two genome-wide significant QTLs on chromosome 5 that influenced locomotor activity (distance, m). The first QTL was for Day 2 distance traveled (saline) and was localized more proximally on chromosome 5 (peak = 22 cM [41 Mb]; LOD = 3.8; *p* = 0.037; Bayes: 14–34 cM [29–66 Mb]; 10% of the phenotypic variance explained Figure 3A,B). A complete list of SNPs, indels and SVs for the Day 2 QTL was obtained from the Sanger database (https://www.sanger.ac.uk/)^33,35^ and is provided in Table S1. The second QTL was for distance traveled D3 following 2 mg/kg methamphetamine and was more distally localized near the *Gabra2* intronic deletion (71 Mb) (peak = 35 cM [67 Mb]; LOD = 5.2; *p* < 0.001; Bayes interval: 32–47 cM [60–95 Mb]; 14% of the phenotypic variance explained; Figure 3A,B). A similarly localized QTL was also detected on D4 following the second methamphetamine injection (peak = 34 cM [66 Mb]; LOD = 3.6; *p* = 0.034; Bayes: 16–46 cM [30–93 Mb]; 12% of the phenotypic variance explained; Figure 3A,B). A complete list of SNPs, indels and SVs for the Day 3 (the most robust methamphetamine QTL) was obtained from the Sanger database (https://www.sanger.ac.uk/)^33,35^ and is provided in Table S2. The effect plot of total distance traveled for D1 through D5 over 30 min at the peak associated marker (rs29547790; 70.93 Mb) indicated that the B6J allele was associated with increased methamphetamine-induced distance traveled (Figure 3C). In examining the time course for the chromosome 5 QTL for the sex-combined data set, the J allele was consistently associated with increased methamphetamine-induced distance traveled across the six, 5-min time bins (Figure 3D). RM ANOVA indicated a main effect of Genotype (F_2,174_ = 7.20; *p* = 0.001), Sex (F_1,174_ = 3.89; *p* = 0.05), Time (F_5,870_ = 485.02; *p* < 2 × 10^−16^), a Genotype × Time interaction (F_10,870_ = 2.39; *p* = 0.0085) but no interactions with Sex (*p*’s > 0.46). There was a significant increase in methamphetamine-induced distance traveled in mice with the J/J allele relative to the J/N and N/N alleles (*Tukey’s *p*_adjusted_ < 0.05 for the three comparisons at each time point; Figure 3D). Because we identified both a Sex × Day interaction and a Substrain × Day interaction in distance traveled in the parental substrains, we broke down and analyzed the time course of the effect plot separately in females and males and found that males clearly drove the QTL effect compared with females (Figure 3E,F). For the females-only data set, RM ANOVA indicated no effect of Genotype (F_2,103_ = 2.13), an effect of Time (F_5,515_ = 290.22; *p* < 2 × 10^−16^) and no interaction (*p* = 0.51).Simple contrasts did not identify any significant differences among genotypes at any time point (*p*’s > 0.05). For males-only, there was a main effect of Genotype (F_2,71_ = 7.49; *p* = 0.0011), Time (F_5,355_ = 195.53; *p* < 2 × 10^−16^) and a Genotype × Time interaction (F_10,355_ = 1.85; *p* = 0.05). Mice with the J/J allele showed a significant increase in methamphetamine-induced distance traveled compared with both J/N and N/N (*Tukey’s *p*_adjusted_ < 0.05 for the three comparisons at each time point; Figure 3F).

Power analysis of the 184 F2 mice that we phenotyped in this study indicated that we had ~80% power to detect QTLs explaining at least 10% of the variance in maximum speed and in distance traveled (Figure S1).

Because there was an age range of the F2 mice and because age can affect both baseline and drug-induced locomotor activity, we also tested for Age as a covariate for the 10 activity-related phenotypes; Age was only a significant covariate for D1 and D2 distance and not for any of the eight other phenotypes (*p*s > 0.4; Table S3). When we re-ran QTL analyses for each of the 10 phenotypes with Age as a covariate (along with Sex), all the existing significant results remained significant except for D2 Distance which was no longer statistically significant (chromosome 5: LOD = 3.1; *p* = 0.10; Table S3). All the nonsignificant results without Age as a covariate remained nonsignificant (Table S3). Thus, none of our fundamental results/conclusions change, regardless of whether Age is included as a covariate.

### 3.4 | Striatal *cis*-eQTL analysis identifies Gabra2 as the top transcript associated with rs29547790; the peak chromosome 5 marker linked to methamphetamine-induced distance traveled

Given that the likely causal variant underlying maximum speed induced by methamphetamine for the chromosome 11 locus was a missense variant in *Cyfip2*,^12^ we turned our attention to the novel chromosome 5 locus whose peak marker (rs29547790;70,931,531 bp) is located just proximally to a functional intronic deletion in *Gabra2* (71 Mb)^13^ which codes for the alpha-2 subunit of the GABA-A receptor. In a genome-wide *cis*-eQTL analysis, we examined eQTLs associated with the peak chromosome 5 marker associated with methamphetamine-induced locomotor activity (rs29547790;70.93 Mb). The top transcript showing an association with rs29547790 was *Gabra2* (chromosome 5, 70.96 Mb; *p*_adjusted_ = 7.24 × 10^−27^; Table 1; see Table S4 for a complete list of cis-eQTLs). Given this finding and the prior literature implicating *Gabra2* in psychostimulant behavioral responses,^54^ *Gabra2* is a top candidate quantitative trait gene underlying the chromosome 5 QTL for methamphetamine-induced locomotor activity.

Cis-eQTLs typically explain a much greater proportion of the variance compared with rodent behavioral QTLs and therefore, much smaller sample sizes are sufficient for cis-eQTL analysis.^21,22^ In examining the cumulative proportion of cis-eQTLs versus the percent variance explained in gene expression, we found that of the 1516 cis-eQTLs that were detected (FDR < 0.05; Table S4), ~75% of these cis-eQTLs explained between 15% and 30% of the variance (Figure S2). Notably, the Gabra2 eQTL explained 72% of the variance in Gabra2 expression (Figure S2).

### 3.5 | *Gabra2*-correlated transcripts and enrichment analysis

To gain insight into the neurobiological adaptations associated with differential *Gabra2* expression at the genomic level, we examine the correlation of Gabra2 with other transcripts genome-wide. We identified 148 transcripts with an absolute Pearson’s r value of 0.5 or greater (*p* ≤ 0.015; Table S5). We conducted enrichment analysis of this gene list using Enrichr^52,53^ to identify GO molecular, cellular and biological functions linked to decreased *Gabra2* expression. We identified several enrichment terms related to the GABA-A receptor signaling (Table 2) that were mainly driven by other GABA-A receptor subunits that were positively correlated with *Gabra2* expression (*Gabre*, *Gabrg3*; *r* = +0.56, +0.50; *p* ≤ 0.015; Table S6). Other nominally, positively correlated GABA-A subunit transcripts included Gabrb1 (*r* = +0.37; *p* = 0.08) and Gabrq (*r* = +0.36; *p* = 0.09) (Table S6). Additional GABA signaling-relevant transcripts that were correlated include the GPCR kinase coded by *Grk5* (*r* = +0.51; *p* = 0.013)^55^ and the amino acid transporters coded by *Slc3a2* (*r* = −0.51; *p* = 0.014) and *Slc38a5* (*r* = −0.59; *p* = 0.003)^56^ (Table 3).

### 3.6 | CRISPR/Cas9 correction of the functional intronic deletion in *Gabra2* reduces methamphetamine-induced distance traveled in B6J mice toward a B6NJ-like level

Based on co-mapping of a behavioral QTL with a highly robust striatal cis-eQTL for *Gabra2* expression and based on the known functional intronic variant within *Gabra2* harbored by the B6J substrain that decreases Gabra2 expression,^13^ we tested the hypothesis that this single intronic nucleotide deletion in *Gabra2* located near a splice acceptor site (71,014,638 bp) in the B6J strain is the QTV underlying variance in methamphetamine-induced locomotor activity. Specifically, we predicted that the *Gabra2* deletion increases methamphetamine sensitivity in B6J mice and that correction of this deletion via CRISPR-Cas9-mediated insertion of the B6NJ wild-type nucleotide would reduce methamphetamine-induced distance traveled toward a B6NJ-like level. Furthermore, given the more pronounced phenotypic effect of Genotype at the *Gabra2* locus in males compared with females (Figure 3E,F), we predicted that genotypic restoration of the wild-type allele would exert a more pronounced decrease in methamphetamine-induced distance traveled in male versus female mice.

**FIGURE 4:**
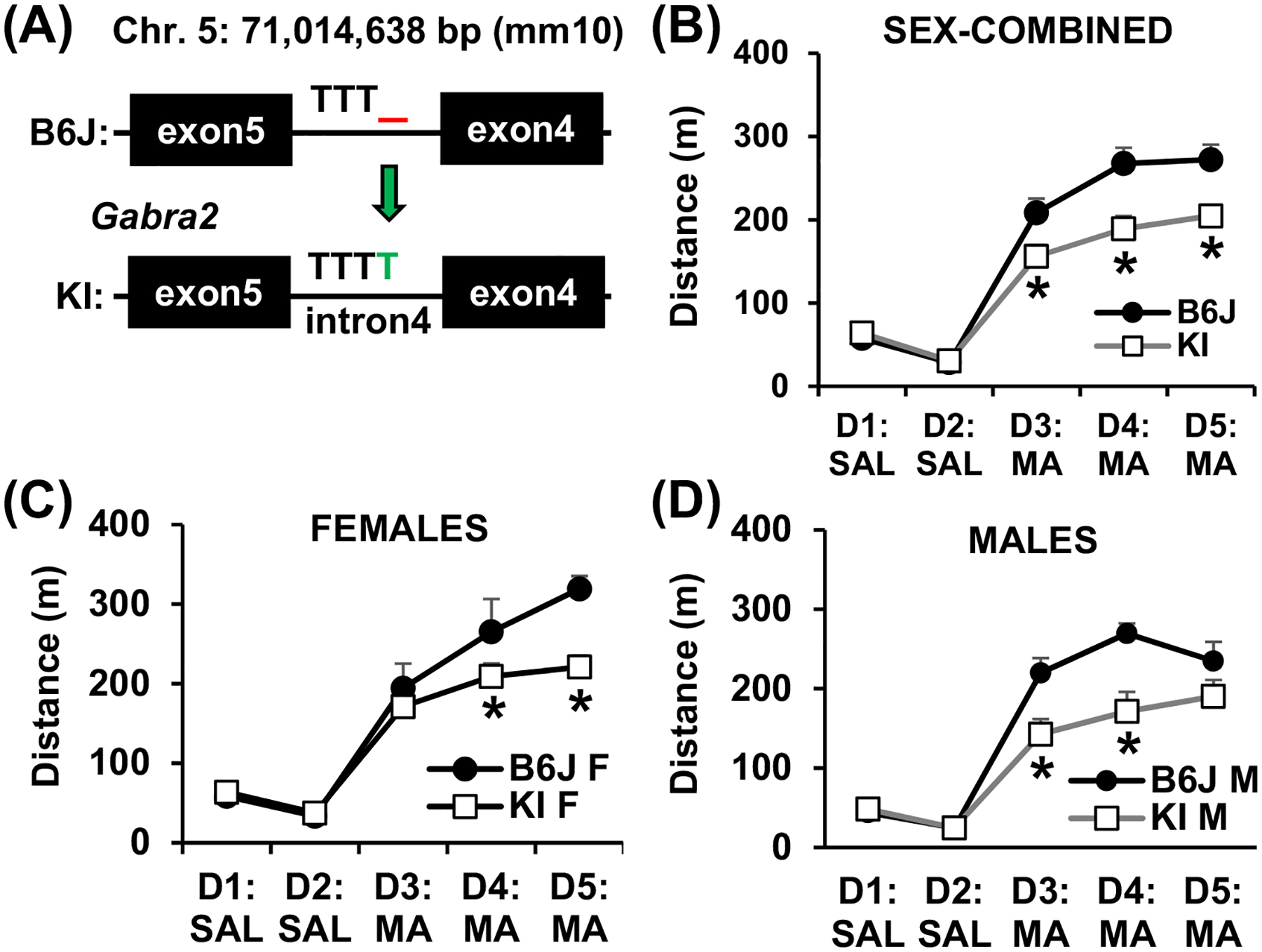
Identification of a quantitative trait variant in *Gabra2* that underlies variation in methamphetamine stimulant sensitivity as measured via distance traveled. (A) Schematic of gene-edited knockin (KI) of the single T nucleotide “corrected” allele that was inserted into intron 4 of the *Gabra2* gene. The C57BL/6J (B6J) substrain harbors a single nucleotide T deletion on chromosome 5 at 71,041,638 bp (mm10). CRISPR-Cas9 was used to insert the deleted T nucleotide onto the B6J genome thus “correcting” the single nucleotide deletion. (B) Distance traveled across Day(D) 1 through D5 in *Gabra2* KI versus B6J wild-types. Simple contrasts of the sex-combined data showed a significant decrease in distance traveled in KI versus B6J mice on D3 (*p = 0.0063), D4 (*p < 0.0001) and D5 (*p = 0.0001). (C) Distance traveled in females. Simple contrasts identified a significant decrease in distance traveled in *Gabra2* KI females versus B6J wild-type females on D4 (*p = 0.0498) and D5 (*p = 0.0009). (D) Distance traveled in males. Simple contrasts showed a significant decrease in distance traveled in KI males versus B6J wild-type males on D3 (*p = 0.0016) and D4 (*p = 0.0001), but not on D5 (*p* = 0.061)

Consistent with our predictions, there was no genotypic difference in distance traveled on Day 1 or Day 2 following saline (i.p.) injections. Sex-combined data from C57BL/6J mice homozygous for the Gabra2 mutational correction (reinsertion of the deleted intronic nucleotide; “knockin” [***Gabra2* KI**]; Figure 4A) showed a decrease in methamphetamine-induced distance traveled on all 3 days of methamphetamine exposure (D3, D4, D5; Figure 4B). RM ANOVA of the sex-combined data showed a main effect of Genotype (F_1,31_ = 9.63, *p* = 0.0041), Day (F_4,124_ = 177.28, *p* < 2 × 10^−16^) and a Genotype × Day interaction (F_4,124_ = 7.36, *p* = 2.37 × 10^−5^). There was no statistically significant effect of Sex (F_1,31_ = 2.77; *p* = 0.11). Furthermore, the interaction of Sex with Day (F_4,124_ = 2.25; *p* = 0.068) and the Genotype × Sex × Day interaction (F_4,124_ = 2.09; *p* = 0.087) did not reach statistical significance. Simple contrasts of the sex-combined data showed a significant decrease in distance traveled in KI versus B6J mice on D3 (*p = 0.0063), D4 (*p < 0.0001) and D5 (*p = 0.0001) (Figure 4B). Consistent with the QTL results from F2 mice at the chromosome 5 locus, there was no genotypic difference in methamphetamine-induced locomotor activity in females on Day 3 (Figure 4C), while on Day 4 and Day 5, female KI mice showed a significant decrease in methamphetamine-induced locomotor activity (Figure 4C). RM ANOVA of the females-only data set indicated a main effect of Day (F_4,56_ = 73.86, *p* < 2 × 10^−16^) and a Genotype × Day interaction (F_4,56_ = 3.38, *p* = 0.015). Simple contrasts identified a significant decrease in distance traveled in *Gabra2* KI females versus B6J wild-type females on D4 (*p = 0.0498) and D5 (*p = 0.0009) (Figure 4C). On the other hand, and again, consistent with the QTL results from F2 mice, male KI mice showed a significant decrease in methamphetamine-induced distance traveled following the first and second methamphetamine administration on Day 3 and Day 4, with no genotypic difference on Day 5 (Figure 4D). RM ANOVA of males-only indicated a main effect of Genotype (F_1,17_ = 6.49, *p* = 0.021), Day (F_4,68_ = 109.21, p < 2 × 10^−16^) and a Genotype × Day interaction (F_4,68_ = 6.60, *p* = 0.00015). Simple contrasts showed a significant decrease in distance traveled in KI males versus B6J wild-type males on D3 (*p = 0.0016) and D4 (*p = 0.0001), but not on D5 (*p* = 0.061) (Figure 4D).

**FIGURE 5:**
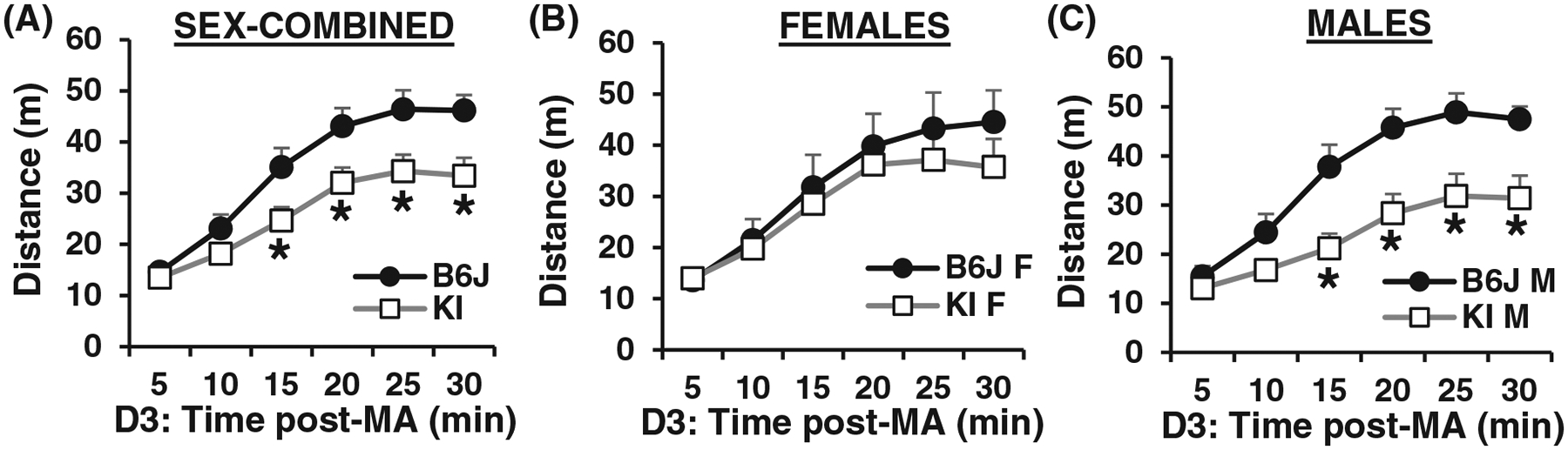
Time course of distance traveled following acute methamphetamine administration on Day 3 in *Gabra2* KI mice versus B6J wild-types. (A) Distance traveled on Day(D) 3 across 5-min bins in KI versus B6J wild-types. Simple contrasts showed a significant decrease in distance traveled at 15 min (*p* = 0.022), 20 min (*p* = 0.016), 25 min (*p* = 0.0078) and 30 min (*p* = 0.0046). (B) Time course of distance traveled in females on D3 following acute methamphetamine administration. (C) Time course of distance traveled in males on D3 following acute methamphetamine administration. Simple contrasts showed a significant decrease in KI males versus B6J wild-type males at 15 min (*p = 0.0027), 20 min (*p = 0.0017), 25 min (*p = 0.0021) and 30 min (*p = 0.0034)

Based on the QTL that was significant for the first methamphetamine exposure on Day 3 (Figure 3A,B; green traces), we were primarily interested in this phenotype and so we broke down the data for Day 3 into 5-min bins like we did with the F2 mice (Figure 3D–F) to more closely examine the sex-combined and sex-stratified data sets. Similar to the F2 results for the chromosome 5 locus, the effect of Genotype on acute methamphetamine-induced locomotor activity was more pronounced in males. For the sex-combined data set, *Gabra2* KI mice showed a significant decrease in methamphetamine-induced locomotor activity from 10 to 30 min post-methamphetamine (Figure 5A). RM ANOVA showed a main effect of Genotype (F_1,31_ = 5.723, *p* = 0.023), Time (F_5,155_ = 96.60, *p* < 2 × 10^−16^) and a Genotype × Time interaction (F_5,155_ = 4.27, *p* = 0.0011). Simple contrasts showed a significant decrease in distance traveled at 15 min (*p* = 0.022), 20 min (*p* = 0.016), 25 min (*p* = 0.0078) and 30 min (*p* = 0.0046) (Figure 5A). For females-only, there was no significant genotypic difference at any of the six time bins (Figure 5B). RM ANOVA of the females-only data set indicated a main effect of Time (F_5,70_ = 31.74, *p* < 2 × 10^−16^) but no effect of Genotype (F_1,14_ < 1) and no Genotype × Time interaction (F_5,70_ < 1) (Figure 5B). For males, there was a significant decrease in methamphetamine-induced locomotor activity in KI males from 15 to 30 min (Figure 5C). RM ANOVA of males-only indicated a main effect of Genotype (F_1,17_ = 8,32, *p* = 0.01), Time (F_5,85_ = 78.78, *p* < 2 × 10^−16^) and a Genotype × Time interaction (F_5,85_ = 6.53, *p* = 3.5 × 10^−5^). Simple contrasts showed a significant decrease in KI males versus B6J wild-type males at 15 min (*p = 0.0027), 20 min (*p = 0.0017), 25 min (*p = 0.0021) and 30 min (*p = 0.0034) (Figure 5C). Thus, like F2 males with the chromosome 5 QTL, male *Gabra2* KI mice account for the sex-combined phenotype of decreased methamphetamine-induced distance traveled in mice with the wild-type (KI) allele compared with the *Gabra2* deletion.

Because of the age range of mice in this experiment (86–155 days old), we tested for Age as a potential covariate in an analysis of covariance (ANCOVA) model. We found no significant effect of Age (F_1,27_ = 0.25; *p* = 0.62), no Genotype × Age interaction (F_1,27_ = 0.26; *p* = 0.61), no Sex × Age interaction (F_1,27_ = 0.48; *p* = 0.50), no, Genotype × Sex × Age interaction (F_1,27_ = 0.35; *p* = 0.56), no Day × Age interaction (F_4,108_ = 0.16; *p* = 0.96), no Day × Genotype × Age interaction (F_4,108_ = 1.0; *p* = 0.41), no Day × Sex × Age interaction (F_4,108_ = 0.20; *p* = 0.94) and no Day × Strain × Sex × Age interaction (F_4,108_ = 1.77; *p* = 0.14).

## 4 | DISCUSSION

We replicated the enhanced psychostimulant sensitivity in B6J compared with N substrains^12^ (Figure 1). We then used a Reduced Complexity Cross between C57BL/6 substrains to identify a QTL near the *Cyfip2* missense mutation previously identified for cocaine velocity^12^ that influenced methamphetamine-induced maximum speed (Figure 2). Next, we identified a novel QTL near the known functional intronic deletion in *Gabra2*^13^ that influenced methamphetamine-induced distance traveled (Figure 3). *Cis*-eQTL analysis of striatal tissue from F2 mice implicated *Gabra2* as a causal quantitative trait gene underlying increased methamphetamine stimulant sensitivity (Table 1). Finally, CRISPR/Cas9 correction of the single nucleotide deletion via CRISPR-Cas9 reversed the enhanced methamphetamine sensitivity toward a C57BL/6NJ-like level (Figure 4), thus recapitulating the QTL effect (Figure 5 vs. Figure 3) and identifying the quantitative trait nucleotide.

Despite the fact that Gabra2 KI mice recapitulated the effect of Sex-dependent effect of Genotype at the chromosome 5 QTL containing the *Gabra2* deletion, neither result recapitulated the parental strain phenotype where both female B6J and male B6J mice harboring the *Gabra2* deletion showed qualitatively similar increases in methamphetamine-induced distance traveled on Day 3 (Figure 1E,F). This discrepancy is likely explained by the fact that these B6 substrains each harbor their own unique set of variants that contribute to the overall phenotype, including notably, the *Cyfip2* missense mutation in C57BL/6N that decreases psychostimulant velocity.^12^

To our knowledge, this is the first time that a *Gabra2* variant has been identified to influence methamphetamine behavior as previous studies focused primarily on cocaine, for example, *Gabra2* knockouts with deletion of exon 4.^57^ *Gabra2* encodes the alpha 2 subunit of the gamma-aminobutryic acid A (GABA-A) receptor, a pentameric, ligand-gated ion channel that mediates fast inhibitory neurotransmission via ligand-gated choloride influx and neuronal hyperpolarization.^58^ Gabra2-containing GABA-A receptors are expressed in several limbic regions involved in motivation and reward, including notably, the nucleus accumbens.^46,47^ Chronic methamphetamine administration leads to an increase in Gabra2 mRNA in several brain regions, including the striatum and ventral tegmental area.^59^ Furthermore, *GABRA2* variants are associated with variance in psychostimulant behavioral responses and addiction, including cocaine^60–62^ and methyphenidate.^63^

Interestingly, in contrast to our observation of enhanced methamphetamine behavior in B6J mice with the *Gabra2* intronic indel (corresponding to reduced Gabra2 expression), constitutive *Gabra2* knockout mice on a mixed C57BL/6J/129SvEv genetic background showed a decrease in cocaine-induced behaviors, including cocaine-induced reinforcement and locomotor sensitization,^60^ with no phenotypic difference in cocaine IVSA or reinstatement of cocaine seeking.^64^ Discrepancies could potentially be explained by multiple factors. First, the *Gabra2* intronic deletion is a different type of mutation compared with deletion of an entire exon (exon 4) in *Gabra2* knockouts^57^—there is still a clearly detectable level of expression at the mRNA and protein level in multiple brain regions in B6J mice containing the *Gabra2* intronic indel.^13^ Thus, there could be fewer or different compensatory neuroadaptations in the GABA system (e.g., changes in other GABA-A subunits) in response to the constitutive *Gabra2* intronic indel compared with the *Gabra2* knockout. Interestingly, our findings are consistent with some of the literature. For example, like the *Gabra2* intronic indel, antisense oligodeoxynucleotides against *Gabra2* in the striatum adult Sprague–Dawley rats increased sensitivity to cocaine-induced locomotor activity and stereotypy.^65^ Second, methamphetamine has a different mechanism of action (reverses transport of monoamines) than cocaine (blocks transport of monoamines) and thus, it is possible that the *Gabra2* variants lead to different effects with different psychostimulants.^66^ In support, the *Gabra2* locus was not identified to be linked to cocaine velocity in a reduced complexity cross that segregated this variant.^12^ To our knowledge, behavioral responses to amphetamines have not been reported in *Gabra2* knockouts. Third, because the *Gabra2* knockout is on a mixed background containing 129SvEv and C57BL/6J alleles, including the *Gabra2* intronic indel, it is possible that phenotypic detection of exon 4 deletion is obscured by segregation of the *Gabra2* intronic indel and/or 129SvEv variants.

The impact of reduced Gabra2 levels on neuronal function has yet to be determined. Dixon and colleagues reported a 33% decrease in miniature inhibitory postsynaptic current (IPSC) and prolonged decay, but no difference in frequency in the nucleus accumbens of *Gabra2* knockout mice.^60^ On the other hand, other groups have reported no change in CA1 pyramidal cell IPSCs following *Gabra2* genetic deletion.^67^ Comparison of perisomatic asynchronous IPSCs in CA1 neurons between B6J containing the *Gabra2* intronic indel and the corrected B6J KI line at baseline and following treatment with a Gabra2/Gabra3 selective positive allosteric modulator (PAM) showed a prolonged decay in the corrected KI line,^68^ indicating a restoration of PAM effects on receptor function. Kearney and colleagues found a reduction in *Gabra2*-containing receptors without a general reduction in overall GABA-A receptors in B6J versus corrected KI mice.^68^ The latter finding implicates a heteromeric receptor composition (containing both *Gabra1* and *Gabra2*) at perisomatic GABAergic synapses and that *Gabra2*-containing receptors might play a major role in mediating perisomatic phasic inhibition. These changes were associated with premature death and more severe seizures in B6J mice heterozygous for deletion of the Dravet syndrome candidate gene *Scn1a* relative to corrected lines heterozygous for the same deletion. However, inhibitory signaling was not profiled in other brain regions. Previous comparisons of GABA-A receptor mRNA expression in cortex, hippocampus and striatum between B6J and the corrected KI line found a more profound alteration in subunit expression in the striatum compared with other brain regions.^13^ Taken together, these results suggest functional alterations in inhibitory signaling associated with a reduction of *Gabra2*, although the precise mechanisms and functional impact are likely to vary by cell type and brain region.

In the context of our previous findings,^13^ our current set of results support the notion that reduced *Gabra2* expression and plausibly, altered pentameric GABA-A receptor function leads to enhanced methamphetamine stimulant sensitivity which is in line with an inverse relationship between GABA-A receptor function (ability to transport chloride) and cocaine-induced locomotor stimulation.^69^ To gain insight into neurobiological adaptations associated with reduced *Gabra2* expression, transcript covariance analysis of striatal tissue from F2 mice identified two other subunits that were positively correlated with Gabra2 expression (*r* ≥ +0.5, *p* < 0.05), including Gabre and Gabrg3 (Table 3; Table S6) and two nominally correlated GABA-A subunit transcripts, including Gabrb1 (*r* = +0.37; *p* = 0.083) and Gabrq (*r* = +0.36; *p* = 0.089) (Table S6). Thus, one hypothesis is that the concomitant decrease in expression in one or more of these GABA-A subunits with Gabra2 leads to decreased assembly and thus a decrease in the number of functional GABA-A receptors. In particular, Gabrg3 and Gabrb1 were highly expressed at a level comparable to Gabra2 (Table S6), providing further confidence in these results. In support of Gabra2-Gabrb1 covariance in expression, a GABRA2 risk variant for alcohol dependence leading to reduced expression of GABRA2 positively correlated with expression of GABRB1, GABRG1 and GABRA4 in human iPSCs.^70^

*Gabrb1* is coexpressed with *Gabra2* in vivo.^54,58^ *GABRB1* variants have been associated with alcohol dependence and co-morbid substance use disorders^54^ and altered fMRI BOLD signal in multiple gyri and the caudate/insula during impulsivity and reward sensitivity tasks;^63^ thus, *GABRB1* is hypothesized to regulate excitability of GABA-A receptors in brain regions underlying reward-related behavior and possibly addiction. Concomitant decreases in Gabrb1 and Gabra2 expression were observed in cortex, striatum and hippocampus of B6J mice relative to the corrected B6J KI line.^13^ In striatum, Gabra2 was previously found to assemble with Gabrb2 or Gabrb3 but not Gabrb1,^71^ which could indicate additional changes in function of GABA-A receptors that do not contain Gabra2. Other subunits that were positively correlated with Gabra2 expression were expressed at a very low levels, including Gabre (*r* = +0.56; *p* = 0.0053) and Gabrq (*r* = +0.25; *p* = 0.089) (Table S5). Thus, these correlations could be spurious.

Interestingly, in both F2 mice and *Gabra2* KI mice, the effect of *Gabra2* Genotype on acute methamphetamine-induced distance traveled was more pronounced in males versus females. The recapitulation of the sex-dependent genotypic effect from the QTL phenotype to the KI phenotype was striking (Figure 3E,F vs. Figure 5B,C) and further strengthens the support for the *Gabra2* indel as the causal, QTV.^13^ What is the mechanism underlying the larger increase in methamphetamine-induced locomotor activity in males with the B6J *Gabra2* intronic deletion? One possibility is that male mutants show a larger reduction in Gabra2 transcript levels. Our present transcriptome data set is not powered to detect sex differences; however, previous data sets fail to support sex-dependent effects of the B6J *Gabra2* indel on Gabra2 expression. We did not identify any previous sex differences in *Gabra2* expression between B6 substrains or in B6J relative to the *Gabra2*-corrected KI line.^13^ Furthermore, using a hypothalamic data set from BXD strains on GeneNetwork that contained both sexes,^72,73^ there was no sex difference in Gabra2 expression in BXD-RI strains with or without the B6J-derived Gabra2 intronic deletion as expression levels in female versus male BXD-Ri strains were highly correlated, regardless of *Gabra2* mutant genotype status (*r* = 0.91; *p* < 1 × 10^−16^; Figure S3). Thus, sex differences in the mutational effect of the B6J-derived *Gabra2* intronic indel on transcription are unlikely to explain the enhanced effect of the chromosome 5 QTL or the *Gabra2* KI allele on methamphetamine sensitivity in males. Alternatively, sex differences in other neurotransmitter or neuromodulatory systems could interact with the *Gabra2* Genotype in determining the methamphetamine behavioral response. For example, sex-dependent interactions of endogenous opioid function and alcohol drinking on Gabra2 expression in mice have been reported.^74^

There are several limitations to this study. First, the parental B6 substrains and the F2 mice used for behavioral QTL analysis all had prior (but equal) exposure to naloxone over the course of 9 days that could have potentially influenced subsequent methamphetamine-induced behaviors as described in the Methods—this turned out not to be the case, as we nicely replicated the B6 substrain difference in methamphetamine-induced locomotor activity that was previously identified for methamphetamine-induced locomotor activity (B6J > B6N).^12^ Prior naloxone exposure could have enhanced our ability to detect the chromosome 5 QTL but this potential confound was offset by the fact that gene-edited *Gabra2* KI mice were completely naïve from any drug or experimental manipulations, yet the results qualitatively recapitulated the effect of the chromosome 5 QTL, despite the fact that the two studies were conducted in entirely different laboratories at different institutions. Another limitation is that the F2 mice from which samples were collected for striatal eQTL analysis had prior exposure to the mu opioid receptor agonist oxycodone as described in the Methods. However, this concern is mitigated by the fact that the eQTL was in the same direction as the prior report of Gabra2 expression differences between B6J and B6N (B6J < B6N).^13,75^ Finally, we only conducted eQTL analysis from a single brain tissue, the striatum. However, note that we previously identified reduced Gabra2 expression at both the transcript and protein levels in multiple brain regions, including cortex, hippocampus and striatum^13^; thus, the functional effects of this variant on Gabra2 expression are ubiquitous across CNS tissues examined.

To summarize, we replicated a historical QTL near the *Cyfip2* missense mutation underlying differential sensitivity to psychostimulant-induced maximum speed and identified a novel QTL and QTV in *Gabra2* that underlies enhanced stimulant sensitivity to methamphetamine. This study further illustrates the efficiency of Reduced Complexity Crosses in systems genetic analysis of complex traits to rapidly identify causal genes and nucleotides.^8,9^ Future studies will identify the brain regions, cell types, circuits and physiological mechanisms underlying the relationship between reduced Gabra2 expression and GABA-A receptor function to enhancement of psychostimulant behaviors.

## Supplementary Material

Supplementary Table 1

Supplementary Information

Supplementary Table 2

Supplementary Table 3

Supplementary Table 4

Supplementary Table 5

Supplementary Table 6

## Figures and Tables

**TABLE 1:** Cis-eQTL gene transcripts showing peak marker association in transcript variance with rs29547790 (chromosome 5: 70.9 Mb) (FDR < 0.05)

Gene	Chr.5 location (Mb)	Distance from rs29547790 (Mb)	Log2FC (JJ vs. NN)	*p*-value	Adjusted *p*-value
Gabra2	70.96	0.026	−0.88	1.66E–30	7.24E–27
Muc3a	137.21	66.28	0.56	2.60E–10	1.14E–07
Atp8a1	67.62	3.08	0.18	1.28E–06	9.57E–05
Ptpn11	121.13	50.20	0.16	2.17E–05	0.000778
Cxcl5	90.76	19.83	−2.41	3.22E–05	0.001042
Fgfr3	33.72	37.19	0.19	0.000123	0.002843
Cux2	121.86	50.92	−0.33	0.000136	0.003049
Ankrd13a	114.77	43.84	0.27	0.00029	0.005321
Whsc1	33.82	37.03	−0.14	0.000399	0.006869
Mn1	111.42	40.49	0.24	0.000644	0.009812
Auts2	131.44	60.51	0.20	0.000721	0.01069
Cds1	101.77	30.83	−0.09	0.002214	0.02399
Lrrc8c	105.52	34.59	0.22	0.002975	0.0293
Tsc22d4	137.75	66.81	0.04	0.005113	0.043141
Garem2	30.11	40.81	0.23	0.005705	0.046604

*Note*: Red rows = decreased expression with the C57BL/6J allele versus the C57BL/6NJ allele; blue rows = increased expression with the C57BL/6J allele versus the C57BL/6NJ allele.

**TABLE 2:** Enrichment analysis of genes correlated with Gabra2 transcript levels in F2 mice (*n* = 23; *r* ≤ −0.5 or *r* ≥ +0.5; *p* ≥ 0.015)

GO: Biological process							
Term	GO #	Overlap	*p*-value	Adj. *p*	Z-score	Combined Score	Genes
Synaptic transmission, GABAergic	51,932	3/12	7.83E–05	0.05354	−2.45	23.13	GABRA2;GABRE;GABRG3
GABA signaling pathway	7214	3/20	3.89E–04	0.132938	−2.06	16.21	GABRA2;GABRE;GABRG3
L-amino acid transport	15,807	3/26	8.59E–04	0.195793	−2.22	15.68	PRAF2;SLC3A2;SLC38A5
Regulation of postsynaptic membrane potential	60,078	3/34	1.89E–03	0.257775	−1.51	9.49	GABRA2;GABRE;GABRG3
Chemical synaptic transmission, postsynaptic	99,565	3/34	1.89E–03	0.257775	−1.45	9.09	GABRA2;GABRE;GABRG3
GO: Cellular component							
Term	GO #	Overlap	*p*-value	Adj. *p*	Z-score	Combined Score	Genes
GABA-A receptor complex	1,902,711	3/20	3.89E–04	0.023457	−2.19	17.20	GABRA2;GABRE;GABRG3
dendrite membrane	32,590	3/21	4.51E–04	0.023457	−2.78	21.38	GABRA2;GABRE;GABRG3
germ plasm	60,293	2/14	4.49E–03	0.123421	−2.36	12.75	TDRD1;SNRPG
Cytoskeleton	5856	10/521	4.75E–03	0.123421	−1.58	8.45	BCAS3;ACTR1A;EDA;MYOT; MVP;SHROOM2;MYOZ3; NPM3;S100A9;MDN1
P granule	43,186	2/17	6.61E–03	0.137495	−2.35	11.81	TDRD1;SNRPG
GO: Molecular function							
Term	GO #	Overlap	*p*-value	Adj. *p*	Z-score	Combined Score	Genes
Benzodiazepine receptor activity	8503	3/11	5.90E–05	0.009565	−2.81	27.34	GABRA2;GABRE;GABRG3
GABA-gated chloride ion channel activity	22,851	3/13	1.01E–04	0.009565	−2.49	22.93	GABRA2;GABRE;GABRG3
Inhibitory extracellular ligand-gated ion channel activity	5237	3/16	1.95E–04	0.012288	−2.39	20.38	GABRA2;GABRE;GABRG3
Ligand-gated anion channel activity	99,095	3/19	3.32E–04	0.014028	−2.16	17.29	GABRA2;GABRE;GABRG3
GABA-A receptor activity	4890	3/20	3.89E–04	0.014028	−2.09	16.42	GABRA2;GABRE;GABRG3

**TABLE 3:** Pearson’s *r* for genes identified from enrichment analysis (see Table 2) that were correlated with Gabra2

Gene	Name	Chr	Start	*r*	*p* value
**Gabra2**	**Gamma-aminobutyric acid (GABA) A receptor, subunit α2**	5	70.96	1.00	0
Myot	Myotilin	18	44.33	0.63	0.001
Hba-a1	Hemoglobin alpha, adult chain 1	11	32.28	−0.62	0.002
Eda	Ectodysplasin-A	X	99.98	0.62	0.002
Npm3	Nucleoplasmin 3	19	45.75	0.59	0.003
Slc38a5	Solute carrier family 38, member 5	X	8.27	−0.59	0.003
Mdn1	Midasin AAA ATPase 1	4	32.66	0.56	0.005
**Gabre**	**Gamma-aminobutyric acid (GABA) A receptor, subunit ϵ**	X	72.23	0.56	0.005
Shroom2	Shroom family member 2	X	152.6	−0.56	0.005
Myoz3	Myozenin 3	18	60.57	0.56	0.006
Snrpg	Small nuclear ribonucleoprotein polypeptide G	6	86.37	0.56	0.006
Hba-a2	Hemoglobin α, adult chain 2	11	32.3	−0.55	0.006
Hbb-bs	Hemoglobin, β adult s chain	7	103.8	−0.53	0.009
S100a9	S100 calcium binding protein A9	3	90.69	−0.53	0.01
Actr1a	ARP1 actin-related protein 1A, centractin α	19	46.38	−0.52	0.01
Mvp	Major vault protein	7	127	−0.52	0.011
Bcas3	Breast carcinoma amplified sequence 3	11	85.35	−0.52	0.012
Grk5	G protein-coupled receptor kinase 5	19	60.89	0.51	0.013
Praf2	PRA1 domain family 2	X	7.73	0.51	0.014
Slc3a2	Solute carrier family 3, member 2	19	8.71	−0.51	0.014
Tdrd1	Tudor domain containing 1	19	56.83	−0.50	0.014
**Gabrg3**	**Gamma-aminobutyric acid (GABA) A receptor, subunit γ3**	7	56.72	0.50	0.014

*Note*: Blue rows indicate positive correlation with Gabra2 expression; red rows indicate negative correlation with Gabra2 expression.

## Data Availability

All data in its raw and processed forms will be made immediately available upon request.
