# Supplementary material for: A quantitative trait variant in Gabra2 underlies increased methamphetamine stimulant sensitivity
Source: Genes Brain Behav. Author manuscript; Available in PMC 2022 Jun 1. (PMC9083095; doi:10.1111/gbb.12774)

**SUPPLEMENTARY INFORMATION**

**TABLES**

**Supplementary Table 1 (posted online):** List of variants within the Day 2 QTL for distance traveled following saline (i.p.).

**Supplementary Table 2 (posted online):** List of variants within the Day e QTL for distance traveled following methamphetamine (2 mg/kg, i.p.).

**Supplementary Table 3 (posted online):** Summary of the QTLs with both Age and Sex included as covariates.

**Supplementary Table 4 (posted online):** A complete list of cis-eQTLs (FDR < 0.05).

**Supplementary Table 5 (posted online):** Genes whose transcripts are correlated with Gabra2 expression (r ≤ -0.5 or ≥ + 0.5; p < 0.015).

**Supplementary Table 6:** Correlation of Gabra2 with other transcripts coding for GABA-A receptor subunits.

**FIGURES**

**Supplementary Figure 1 (see below): Power analysis for Day 3 distance traveled in B6J x B6NJ-F2 mice.** Power versus effect size (% variance explained) for an additive QTL model (no covariates) and a sample size of 184 F2 mice. 0.2, 0.4, 0.6, and 0.8 power is achieved with an observed effect size of 3.86%, 5.65%, 7.41%, and 9.65% variance explained, respectively.

**Supplementary Figure 2 (see below): Cumulative proportion of *cis*-eQTLs versus percent variance explained in gene expression.**

**Supplementary Figure 3 (see below): Correlation of Gabra2 expression in female versus male mice from various BXD-RI substrains with or without the *Gabra2* intronic indel.** Plotted number indicate the BXD-RI strain. BXD-RI strains with the mutant Gabra2 allele include 40, 44, 60, 83, and 89. X and Y-axes indicate the dataset from GeneNetwork. The hypothalamus was selected for this analysis because it had both females and males contained within the dataset.

**Supplementary Figure 1**


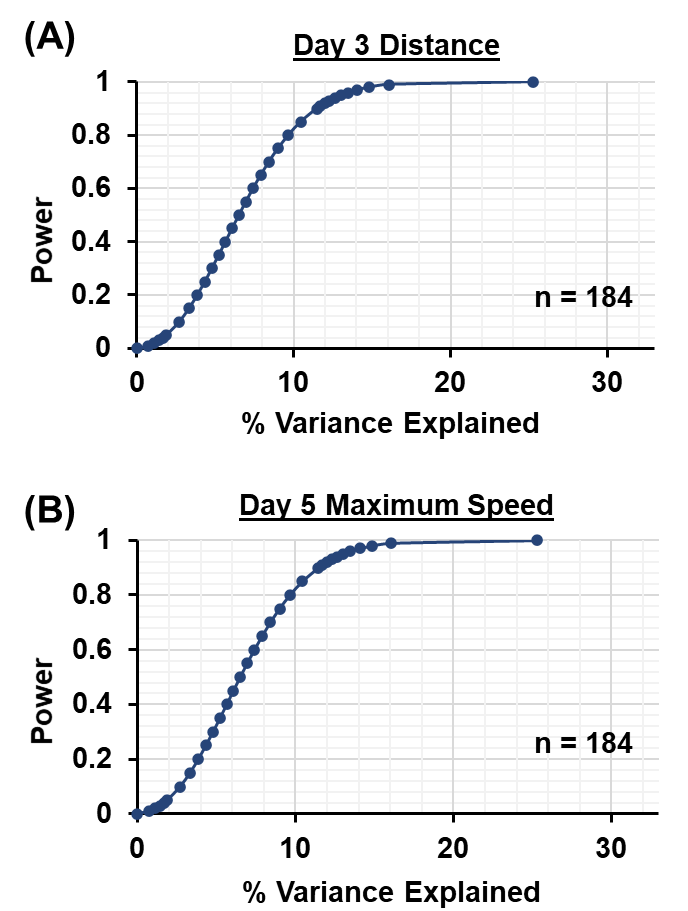


**Supplementary Figure 2**


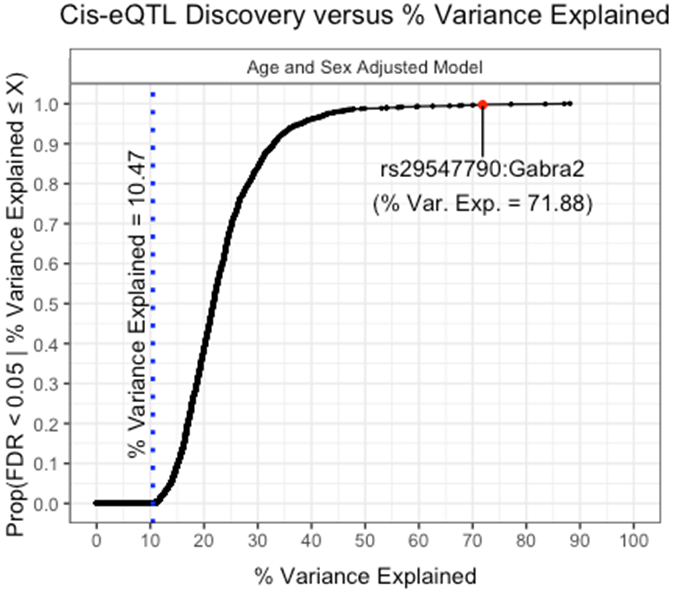


**Supplementary Figure 3**


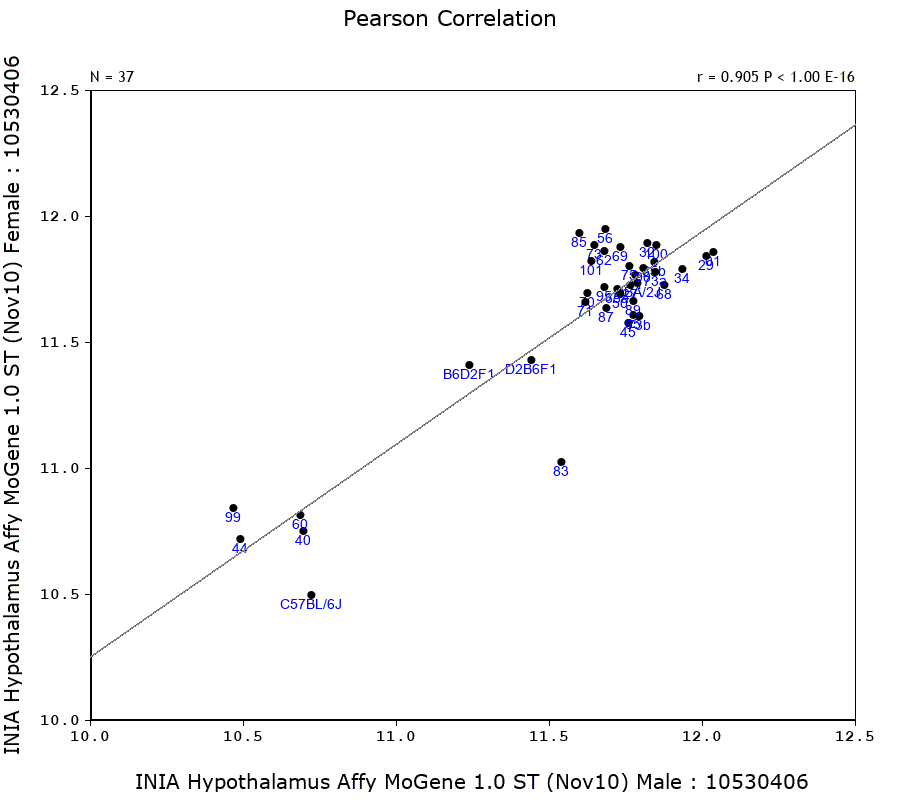

Supplement: Supplementary Information [file NIHMS1803240-supplement-Supplementary_Information.docx]
